# Key Proteins for Regeneration in *A. mexicanum*: Transcriptomic Insights From Aged and Juvenile Limbs

**DOI:** 10.1155/2024/5460694

**Published:** 2024-11-14

**Authors:** Aylin Del Moral-Morales, Cynthia Sámano, José Antonio Ocampo-Cervantes, Maya Topf, Jan Baumbach, Jossephlyn Hernández, Karla Torres-Arciga, Rodrigo González-Barrios, Ernesto Soto-Reyes

**Affiliations:** ^1^Departamento de Ciencias Naturales, Universidad Autónoma Metropolitana-Cuajimalpa (UAM-C), Mexico City, Mexico; ^2^Centro de Investigaciones Biológicas y Acuícolas de Cuemanco (CIBAC), Universidad Autónoma Metropolitana-Xochimilco (UAM-X), Mexico City, Mexico; ^3^Centre for Structural Systems Biology (CSSB), Hamburg, Germany; ^4^Leibniz-Institut für Virologie (LIV), Hamburg, Germany; ^5^University Medical Center Hamburg-Eppendorf (UKE), Hamburg, Germany; ^6^Institute for Computational Systems Biology, University of Hamburg, Hamburg, Germany; ^7^Computational BioMedicine Lab., University of Southern Denmark, Odense, Denmark; ^8^Unidad de Investigación Biomédica en Cáncer, Instituto Nacional de Cancerología-Instituto de Investigaciones Biomédicas, UNAM, Mexico City, Mexico

**Keywords:** aging, *Ambystoma mexicanum*, blastema, tissue regeneration, transcriptomics

## Abstract

The axolotl, known for its remarkable regenerative abilities, is an excellent model for studying regenerative therapies. Nevertheless, the precise molecular mechanisms governing its regenerative potential remain uncertain. In this study, we collected samples from axolotls of different ages, including 8-year-old individuals and 8-month-old juveniles, obtaining their blastemas 10 days after amputation. Subsequently, we conducted a transcriptomic analysis comparing our samples to a set of previously published experiments. Our analysis unveiled a distinctive transcriptional response in the blastema, characterized by differential gene expression associated with processes such as bone and tissue remodeling, transcriptional regulation, angiogenesis, and intercellular communication. To gain deeper insights, we compared these findings with those from aged axolotls that showed no signs of regeneration 10 days after amputation. We identified four genes—*FSTL1*, *ADAMTS17*, *GPX7*, and *CTHRC1*—that showed higher expression in regenerating tissue compared to aged axolotls. Further scrutiny, including structural and homology analysis, revealed that these genes are conserved across vertebrate species. Our discoveries point to a group of proteins relevant to tissue regeneration, with their conservation in vertebrates suggesting critical roles in development. These findings also propose a novel gene set involved in axolotl regeneration, laying a promising foundation for future investigations across vertebrates.

## 1. Introduction

The axolotl is a salamander of the genus *Ambystoma*, which comprises 32 species from southern Canada to central Mexico. Among the different species present in Mexico, *Ambystoma mexicanum*, an endemic species from the lake region of Mexico City, is the most studied [1, 2]. Commonly known as axolotls, *A. mexicanum* is a vertebrate amphibian with an unusual appearance. Unlike other salamanders, axolotls do not metamorphose to become adults; instead, they remain in their larval form, retaining their external gills and aquatic lifestyle throughout their entire lives [3, 4]. In addition to their popularity as pets in many parts of the world, this animal is considered an interesting model for studying tissue regeneration. Indeed, they can regenerate a wide range of injured tissues within just a few weeks, including limbs [5, 6], tail, central and peripheral nervous systems [7, 8], iris [9], bone, and muscle [10, 11].

Previous studies have shown that the axolotl regeneration process involves three phases: wound healing, blastema formation, and redevelopment [12, 13]. After an injury, the fibroblasts surrounding the wound acquire proliferative capacity and set the stage for new tissue development [14–17]. Once the wound is closed, the fibroblasts dedifferentiate into mesenchymal progenitor cells that accumulate in an epithelium-covered region called the blastema. It is in this organ where the reconstruction of the missing limb takes place. Blastema cells are thought to have a certain “cellular memory,” as they always differentiate into the same cell type from which they originated, and the new limb will have the same size, shape, and orientation as the amputated one [18–20]. Because of this, the study of the underlying mechanisms and factors involved in blastema regeneration is currently of great interest. Specifically, researchers are interested in dissecting the cellular and molecular events, leading to regeneration in axolotls that could be extrapolated to other species. A previous study demonstrated that mouse growth factors can induce blastema formation in axolotls, suggesting that the machinery required for tissue regeneration may be conserved in mammals [21]. However, the molecular factors involved in the activation of such machinery remain unexplored.

One limitation of studying axolotls is the complexity of their genome; with 32 gigabase pairs of DNA distributed across 14 pairs of chromosomes, it is one of the largest genomes among vertebrates. However, approximately 70% of their genome is composed of repetitive elements, making its assembly a challenge [22]. Furthermore, the lack of a fully annotated transcriptome has hampered comprehensive studies of tissue regeneration, making it difficult to compare the results across publications. Despite these challenges, previous research has identified several candidate genes that may play a role in the regenerative process. However, the mechanistic insights into these genes and how they interact with others are not fully understood [23–25]. Fortunately, recent advances in genome assembly and transcriptome annotation are enabling detailed transcriptomic studies that will shed light on the molecular processes underlying axolotl regeneration [26].

Additionally, the impact of environmental factors, such as habitat, diet, and age, on the regenerative capacity of axolotls has not been investigated [5]. While axolotls can regenerate tissues throughout most of their entire lifespan, this phenomenon becomes less efficient with age [9, 27]. Younger individuals can regenerate tissues in a matter of days or weeks, whereas sexually mature adults may require several months to regenerate a limb [18, 28]. It is also worth noting that most experiments have been carried out on the *d*/*d* strain, a homozygous white mutant lineage established by French naturalists in the mid-19th century. Interestingly, this strain often exhibits regeneration defects due to extensive inbreeding [1, 29]. Therefore, to fully understand the genetic, transcriptomic, and environmental factors involved in tissue regeneration in *A. mexicanum*, and how aging may affect this process [30], it is crucial to study native Mexican axolotl populations from the Xochimilco Lake area.

In this study, we conducted transcriptomic analysis on the limbs of juvenile axolotls (8 months old) and the blastema formed 10 days postamputation, aiming to elucidate the molecular fingerprint of tissue regeneration. Our investigation involved a comparative analysis of our datasets with those previously documented by Bryant in 2017, with the objective of identifying conserved transcriptomic responses in blastemas, even across different axolotl strains [23]. Through a custom annotation of the axolotl proteome, we found that the differentially expressed genes (DEGs) were primarily associated with anatomical development and cell differentiation. Additionally, we collected samples from the limbs of two aged axolotls (8 years old) that did not develop a blastema after amputation. By contrasting the DEGs in blastemas with those in aged limbs, we pinpointed four genes (*FSTL1*, *ADAMTS17*, *GPX7*, and *CTHRC1*) exhibiting heightened expression in regenerating tissues but diminished expression in aged axolotls. These genes were found to be associated with anatomical structure development, as corroborated by structural and conservation analyses, indicating their high conservation across vertebrates and their pivotal roles in development, bone morphogenesis, and cartilage formation in mammals. Our results suggest a set of key axolotl genes that potentially participate in tissue regeneration specifically in juvenile axolotls. These genes could provide a basis for studying tissue regeneration in other vertebrates and hold promise for advancing the field of regenerative medicine.

## 2. Materials and Methods

### 2.1. Sample Collection

All biological samples were sourced from a captive population of Mexican axolotls (*A. mexicanum*) housed at the Unidad de Manejo Ambiental of the Centro de Investigaciones Biológicas y Acuícolas de Cuemanco (UMA-CIBAC), affiliated with the Universidad Autónoma Metropolitana, Xochimilco Campus (UAM-Xochimilco) in Mexico City. This axolotl colony originates from wild individuals captured in the Xochimilco Lake area. Situated adjacent to the lake, CIBAC provides conditions closely resembling the axolotl's natural habitat; the water in their tanks is sourced from the lake, and their diet primarily consists of aquatic worms (*Tubifex*). All procedures were approved and supervised by veterinarians from UMA-CIBAC and UAM-Xochimilco, in accordance with the norms and regulations set by the Mexican Ministry of Environment and Natural Resources (Reference Number CEI.2023.007).

Sedation of the axolotls was achieved through immersion in a tank containing benzocaine at a concentration of 50 mg/L before the amputation process. Biological samples were meticulously collected, using a stereoscopic microscope, from the inferior limbs of five juvenile axolotls (8 months old) as well as from two aged axolotls (8 years old). Specifically, blastema tissues were acquired 10 days postamputation from juvenile axolotls, whereas aged axolotls did not display any development of blastema tissue. Five additional 8-month-old organisms, along with their corresponding blastema generated 10 days after amputation, were collected to perform experimental validation of *ADAMTS-17*. No animals were sacrificed for this study, and the axolotls were safely reintroduced to their habitat at UMA-CIBAC following the final sample collection.

### 2.2. RNA Extraction and Sequencing

The collected tissue was preserved in RNA*later* (Invitrogen) at 4°C for a maximum of 24 h prior to processing. RNA was extracted according to the protocol established by Peña-Llopis and Brugarolas [31]. Its quality and concentration were assessed using the High Sensitivity RNA Tapestation (Agilent Technologies). Ribosomal RNA depletion was performed using the Ribo-Zero Gold Kit (Illumina), and libraries were constructed using the SMARTerStranded V2 kit (Takara Bio). Paired-end (PE) sequencing was carried out on Illumina with a read length configuration of 150 and 20 million reads per sample.

### 2.3. Quantitative Reverse Transcription-Polymerase Chain Reaction (RT-qPCR)

Total RNA from five samples of juvenile axolotl limbs and their blastemas, collected 10 days postamputation, and two samples from aged axolotls were extracted using TRIzol reagent (Invitrogen, Catalog No. 15596026), and RNA integrity and quality were evaluated with TapeStation 2200. cDNA was synthesized through a reverse transcription reaction following the instructions provided by the GeneAmp RNA PCR Core Kit (Applied Biosystem, Catalog No. N8080143) using oligo(dT) primers. qRT-PCR assays were performed on 7500 Real-Time PCR System (4351105, Applied Biosystems, Foster City, CA, USA) using Maxima SYBR Green/ROX qPCR Master Mix (2x) kit (Thermo Scientific, Catalog No. K0222). To assess the expression of *ADAMTS17* and *GAPDH* as the housekeeping gene control, the following oligonucleotides were used: *ADAMTS17* (FW: 5′-CTGCTCTGACCTACAAGTGC-3′, RV: 5′-TTGGCATAAAAGTCTCGGCA-3′) and *GAPDH* (FW: 5′-GCTGCCTCCTATGACGAAAT-3′, RV: 5′-TTCCTCGGTGTATCCCAGAA-3′). The relative expression of *ADAMTS17* was determined using the −△△Ct method, comparing its expression in blastema tissues of juvenile axolotls or limbs of aged axolotls to the control condition and normalized with *GAPDH*. Statistical analyses were performed using a *t*-test in GraphPad 7.

#### 2.3.1. Transcriptomic Analysis

In addition to the experimentally obtained samples, data from two other studies were also analyzed. The accession numbers for the samples used are displayed in Table 1.

Quality assessment of the sequencing data was performed using FastQC [32] Subsequently, raw counts were aligned to the reference genome AmexG_v6.0-DD [26] using STAR v.2.7.9a [33]. The resulting transcript-aligned read counts were quantified using featureCounts for R, and the AmexT_v47 transcriptome, accessible at https://www.axolotl-omics.org/assemblies, was used for this purpose. DESeq2 v.1.32.0 [34] was employed for the differential expression analysis. The tissue obtained after amputation of the juvenile axolotl's limb was used as a control. Two separate comparisons were carried out: aged axolotl limb (8 years old) versus control and 10-day blastema from juvenile axolotls versus control. Genes were designated as DEGs if they exhibited |log2 (fold change)| > 1 and padj < 0.05. For the Bryant dataset, differential expression analysis was conducted for proximal blastema using upper arm tissue as the control and for distal blastema using hand tissue as the control.

#### 2.3.2. Gene Annotation and Protein–Protein Interaction (PPI) Networks

All genes were named according to the “gene_name” field in the AmexT_v47 transcriptome file, accessible at https://www.axolotl-omics.org/assemblies. In cases where the “gene_name” field was either empty or equal to “N/A,” genes were assigned the same name as their gene_id. Priority was given to nomenclature with the [hs] identifier over any other annotation. To distinguish genes with the same name, a numerical identifier was appended; for example, KAZAL.3 indicated the presence of four other genes with the name KAZAL in the dataset. The predicted gene ontology (GO) function and PPIs for each gene were determined using STRING [35]. Only one open reading frame per gene was used as input for STRING; if a gene had more than one transcript, the 0.1 isoform was selected. GO term enrichment analysis was performed using gProfiler2 v.0.2.1 for R [36] with the correction method g:SCS and an adjusted *p* value significance threshold of 0.05. Redundant terms were clustered using rrvgo v.1.4.4 for R [37]. The PPI visualizations were built with KeyPathwayMineR [38] All network visualizations were created with Cytoscape [39].

##### 2.3.2.1. Coexpression Analysis

Our datasets, as well as the ones from Bryant and Caballero-Pérez, were used as input for WGCNA [40]. The raw counts were normalized using variance-stabilizing transformation (VST) from the DESeq2 package. Batch effect correction was performed with Combat from the sva v3.40.0 package for R [41]. The blockwiseModules function was employed to identify coexpression modules within the data. The function was run with a power of 7 and a minimum module size of 30 genes, with the TOMType set to “unsigned.” The resulting coexpression modules were named with random colors. Modules were considered significant if they had a *p* value < 0.05 and |Module-Trait Correlation| > 0.5. GO enrichment analysis for the genes contained in each module was performed with gProfiler2 v.0.2.1 for R^36^, and the PPI network was built with KeyPathwayMineR [38] using only the genes with module membership > 0.7. Visualizations were created with Cytoscape and BioRender.com.

### 2.4. Homology and Structural Biology Analysis

The amino acid sequences for each transcript were obtained from the annotation file (.gtf) for the AmexT_v47 transcriptome. If more than one transcript was available, only the 0.1 isoform was modeled. The amino acid sequence of each protein was used as a query for BLASTP 2.12.0 [42, 43] against the UniProtKB and Swiss-Prot database [44]. Only the sequence with the highest identity percentage in each organism was conserved for the identity plot, and the organisms displayed were selected based on the animal model organisms described in a comprehensive review of model organisms [45]. AlphaFold2 [46] was used to predict the structures of the proteins; templates were used for all predictions, and the ConSurf [47] web server was used to color the residues according to their conservation. Functional domain annotation was performed using the Conserved Domain Database (CDD) [48] and the NCBI Conserved Domain tool [49]. Protein visualizations were created with the PyMOL Molecular Graphics System v.4.60.

## 3. Results

### 3.1. Blastemas Share a Defined Transcriptomic Profile

To explore the dynamics of gene expression during the formation of blastema in *A. mexicanum*, we conducted an experiment involving the RNA sample collection. This was achieved by amputating the right forelimb of five juvenile axolotls (8 months old) at a proximal site. These axolotls were bred and raised within CIBAC, a facility dedicated to maintaining a colony derived from wild specimens captured in Lake Xochimilco. Blastema samples were obtained 10 days after the amputation procedure, and RNA was subsequently extracted. The extracted RNA samples underwent sequencing analysis.

**Figure 1:**
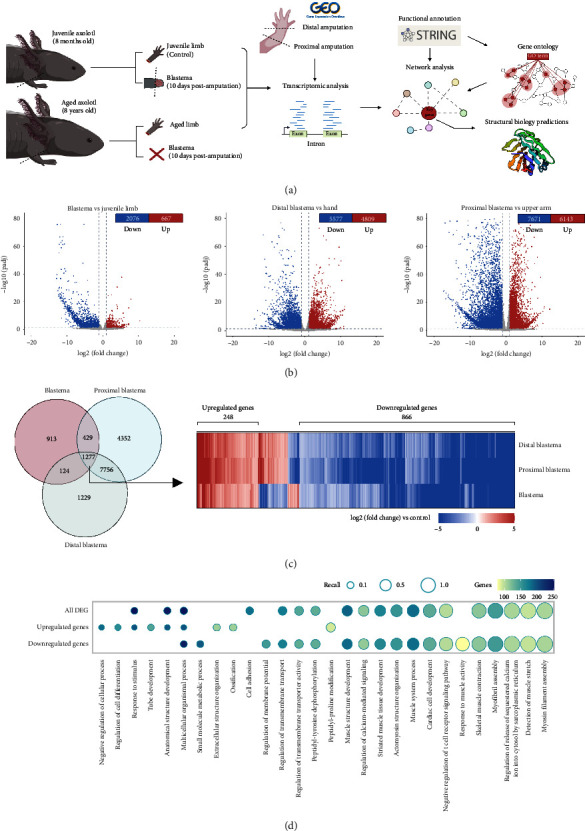
Comparison of DEG in different blastema samples. (a) Schematic representation of tissue sampling and downstream analyses. We sampled five juvenile axolotl limbs (control) and two aged axolotl limbs (over 8 years old). Amputation was performed below the elbow (proximal amputation). Blastemas from juvenile axolotls were collected after 10 days, while aged axolotls showed no blastema at the same time point. Datasets published by Bryant et al. were also analyzed; the samples were taken from *d/d* strain axolotls at two different amputation sites: the first at hand level (distal) and the second below the elbow (proximal). Blastemas from Bryant's dataset were collected 23 days postamputation. After RNA extraction and sequencing, differential expression and network analysis were performed to find relevant genes and predict GO terms associated with regeneration. (b) Differentially expressed gene (DEG) volcano plots. The left panel shows the comparison between blastema and control limbs (juvenile axolotls). The middle panel shows the distal blastema compared to the hand. The right panel shows the proximal blastema compared to the upper arm. Red dots represent the upregulated genes (*p* value < 0.05 and log2 (fold change) > 1). Blue dots represent the downregulated genes (*p* value < 0.05 and log2 (fold change) > 1). (c) Venn diagram of the intersection between DEG for blastema vs. control, distal blastema vs. hand, and proximal blastema vs. humerus. The left panel shows the heat map for the 1277 genes in the intersection between DEG in aged axolotls and blastema. The color of the tiles represents the log2(fold change) value for each gene. Genes with the same behavior across samples are indicated by parentheses (1134 of 1277 genes). d) Top 20 gene ontology biological processes associated with all DEG in the intersection shown in Figure 1(c) (1134 genes), downregulated genes only (886 genes), or upregulated genes (248 genes). The size of the bar corresponds to the number of genes associated with the significant GO term. The color of the bars represents the log10 (*p* value) of the term. Created with BioRender.

To contextualize our findings, we compared them with RNA-seq data from Bryant [23], who studied a strain of d/d axolotls (white mutant). In their study, forelimbs were amputated at two different sites (proximal and distal), and blastemas were collected 23 days postamputation. To identify the most significant changes in gene expression between the different samples, we performed differential expression analysis (Figure 1(a)).

Overall, we observed that most of the DEGs were downregulated. In our samples, we identified 667 upregulated genes and 2076 downregulated genes in the blastema compared to control tissue. In the distal blastema, we found 4809 upregulated genes and 5577 downregulated genes compared to the control, whereas in the proximal blastema, we found 6143 upregulated genes and 7671 downregulated genes (Figure 1(b)).

We compared the DEGs in our samples with Bryant's datasets to search for genes that were consistently regulated across experiments. Our aim was to identify expression patterns associated with regeneration regardless of strain or amputation site. We found 1277 DEGs that were common to all three datasets. Specifically, out of these common DEGs, 248 were upregulated and 886 were downregulated in all three samples (Figure 1(c), Supporting Table 1).

Since ontology annotation for *A. mexicanum* is not available, we used STRING [35] to generate a homology annotation for the coding genes in the *AmexT_v47* transcriptome. By performing a GO term enrichment analysis, we found that the DEGs in the blastema were primarily associated with biological processes related to tissue and muscle development, cytoskeleton organization, cell adhesion, extracellular matrix organization, and ossification. However, among the downregulated genes, we observed an enrichment in biological processes such as cytoskeleton organization, muscle structure development, and myofibril assembly. Conversely, the upregulated genes were enriched in terms such as anatomical structure development, negative regulation of cellular processes, and regulation of cell differentiation (Figure 1(d), Supporting Table 2).

**Figure 2:**
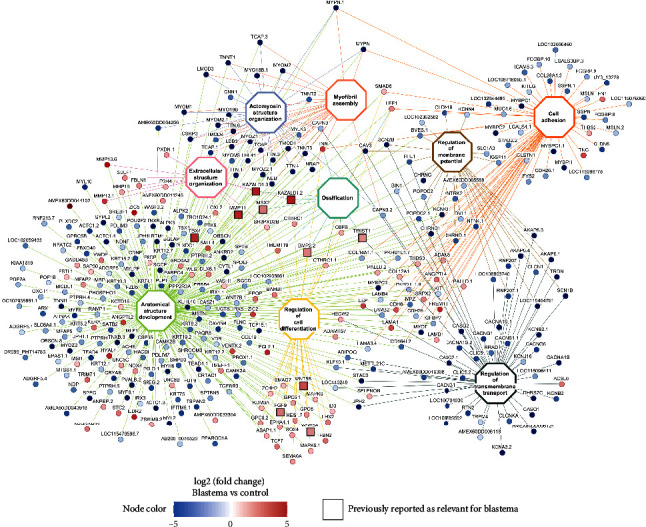
Gene ontology terms and genes associated with axolotl regeneration. Gene ontology terms enriched for the DEG in the three blastema datasets analyzed. Node color corresponds to the log2 (fold change) for the blastema vs. control dataset. GO terms are depicted in white, each with a distinct border color. Edges are colored according to the originating GO term. Squared nodes represent the genes that have been previously reported in the literature to be associated with the axolotl regenerative process. Created with BioRender.

A gene–GO term network was constructed using 10 of the most representative terms from Figure 1(d) to provide a more comprehensive visualization of the genes involved in the process of regeneration. Notably, the majority of genes belonged to the term “anatomical structure development,” and a significant proportion of the genes in this category showed downregulation in the blastema compared to the control. However, genes associated with the Wnt pathway (*WNT5A* and *WNT5B*) were upregulated, as were the metallopeptidases *ADAMTS17*, *ADAM8*, *MMP19*, *MMP11*, and *MMP13*, which are also associated with the “extracellular structure organization” term. Similarly, genes related to cell adhesion, myofibril assembly, and actomyosin structure organization were mostly downregulated in blastemas, with a few exceptions such as tenascin (*TNC*), fibronectin (*FN1*), and thrombospondin-2 (*THBS2*). Another group of genes, including *SMAD6*, *SMAD7*, and *BAMBI*, involved in the tumor growth factor beta (TGF-*β*) signaling pathway, were upregulated and associated with cell differentiation, along with *SOX4* and *NOX4*. Genes associated with epigenetic functions were also found. A notable example is the histone lysine demethylase *KDM3A,* found among the genes involved in differentiation and development. This finding suggests that tissue regeneration may be controlled by epigenetic processes. Notably, genes involved in the ossification and regulation of cell differentiation were also upregulated. Additionally, several genes previously associated with regeneration in axolotls, marked in squared boxes, were obtained from the table published by Haas and Whited [5]. Among these genes, *KAZALD1* stands out as a well-studied gene involved in anatomical development and bone regeneration in axolotls [23]. Furthermore, bone morphogenetic protein 2 (*BMP2*) and fibroblast growth factor 9 (*FGF9*), which are associated with skeletal and cartilage development, were upregulated. These proteins are particularly relevant because a previous study demonstrated their ability to induce blastema formation in axolotls [21]. In summary, our results provide a collection of genes and biological processes that are associated with blastema tissue and the regeneration process in axolotls, which is consistent with and extends the data from previous studies (Figure 2).

#### 3.1.1. Key Genes in Regeneration Revealed by the Transcriptomic Profile of Aged Axolotls

**Figure 3:**
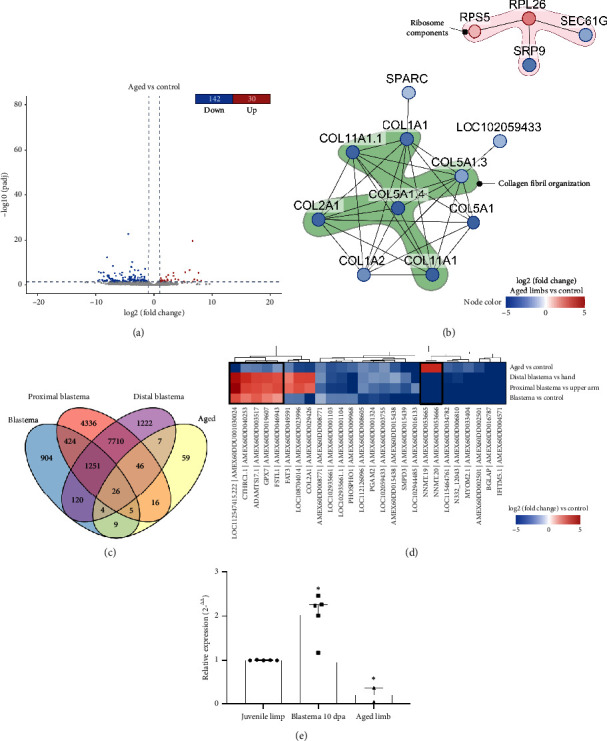
Differential expressed genes in aged axolotls. (a) Volcano plot of the differentially expressed genes (DEGs) obtained for the comparison between aged limbs vs. control limbs (juvenile axolotls). Red dots represent the upregulated genes (*p* value < 0.05 and log2 [fold change] > 1). Blue dots represent the downregulated genes (*p* value < 0.05 and log2 (fold change) < - 1). (b) Protein–protein interaction network for the DEGs in aged limbs. Node color represents the log2 (fold change) of aged limbs vs. control. Genes associated with the term “collagen fibril organization” are highlighted in green, while genes that are part of the ribosome are highlighted in pink. Edges represent the predicted interactions between the proteins encoded by the genes. (c) Venn diagram for the DEGs in all four datasets evaluated. The blastema DEGs correspond to the comparisons made previously in this study. (d) Heat map displaying the fold change of all DEGs in the intersection shown in (c) (26 genes). DEGs that show contrasting patterns between the blastema and aged datasets are highlighted with a black box. (e) Experimental validation of *ADAMTS17* was conducted using qRT-PCR. For this purpose, RNA was extracted from five samples of juvenile axolotl limbs and their respective blastemas, as well as from two aged axolotls. Statistical analyses were performed using a *t*-test. Created with BioRender.

In addition to the previously analyzed samples, we included two aged axolotls (8 years old) in our study, subjecting them to proximal forelimb amputation. Surprisingly, after a period of 10 days postamputation and a subsequent follow-up of 6 months, these aged specimens showed no signs of limb regeneration, in stark contrast to the robust regenerative response observed in juvenile axolotls. Consequently, no postamputation samples were collected from the aged cohort. When we compared the transcriptome of the aged limbs to that of the juvenile limbs, only 172 genes were differentially expressed, with most being downregulated compared to the control (Figure 3(a)). After a GO enrichment analysis, the only significant GO term associated with the DEG in aged limbs was collagen fibril organization.

To investigate relationships among the aged DEGs, we performed *de novo* pathway enrichment using KeyPathwayMineR [38]. As input, we used the DEGs in aged limbs and a PPI network constructed with STRING using the proteome from the *AmexT_v47* annotation file. The interaction network shows mainly the downregulation of type I (*COL1A1, COL1A2*), II (*COL2A1*), V (*COL5A1*), and XI (*COL11A1*) collagens. Some of these proteins appeared more than once because they were assigned the same name in the transcriptome annotation file, so they were marked with a numerical suffix. Additionally, several ribosomal components, such as ribosomal proteins RPS2 and RPS5, as well as signal recognition particle 9 *(SRP9*) and *SEC61G*, which are part of the complex required for protein translation at the endoplasmic reticulum, were observed (Figure 3(b)).

In an effort to identify key genes involved in the tissue regeneration process, we focused on the DEGs that showed contrasting patterns in the blastema vs. control and aged vs. control comparisons. Our goal was to identify genes that were upregulated in blastemas but downregulated in old axolotls, and vice versa, as these could provide valuable insights into the impaired tissue regeneration observed in aging axolotls. Through DEG overlap analysis, we identified 26 genes that consistently exhibited significant differential expression across all four datasets evaluated (Figure 3(c)). Among these, seven genes showed consistent differential expression specifically in blastemas compared to aged limbs. Notably, *CTHRC1.1*, *ADAMTS17*, *GPX7*, *FSTL1*, and *LOC112547415.222* were upregulated in blastemas but downregulated in aged axolotls. Conversely, *NNMT.19* and *NNMT.20* exhibited the opposite pattern, being upregulated in aged axolotls but downregulated in blastemas (Figure 3(d)). In order to experimentally validate one of the key genes involved in tissue regeneration, we analyzed the expression of the *ADAMTS17* gene using limb samples from five 8-month-old juvenile axolotls and their blastema generated 10 days after amputation, as well as samples from 8-year-old aged axolotls. Overexpression of *ADAMTS17* was observed in the blastema of the 8-month-old juvenile axolotls compared to their limb. Interestingly, the expression of this gene significantly decreases in the limbs of 8-year-old aged axolotls compared to those from juvenile axolotls (Figure 3(e)). These genes are of particular interest because their high expression in blastemas, coupled with their downregulation in aged axolotls that have lost their regenerative capacity, suggests that they may play a critical role in the regeneration process. Therefore, we will refer to this set of genes as regeneration-related genes.  Table 2 summarizes the annotation and putative function of these regeneration-related genes.

#### 3.1.2. Coexpression Network Analysis Reveals Gene Modules Associated With Tissue Regeneration in *A. mexicanum*

**Figure 4:**
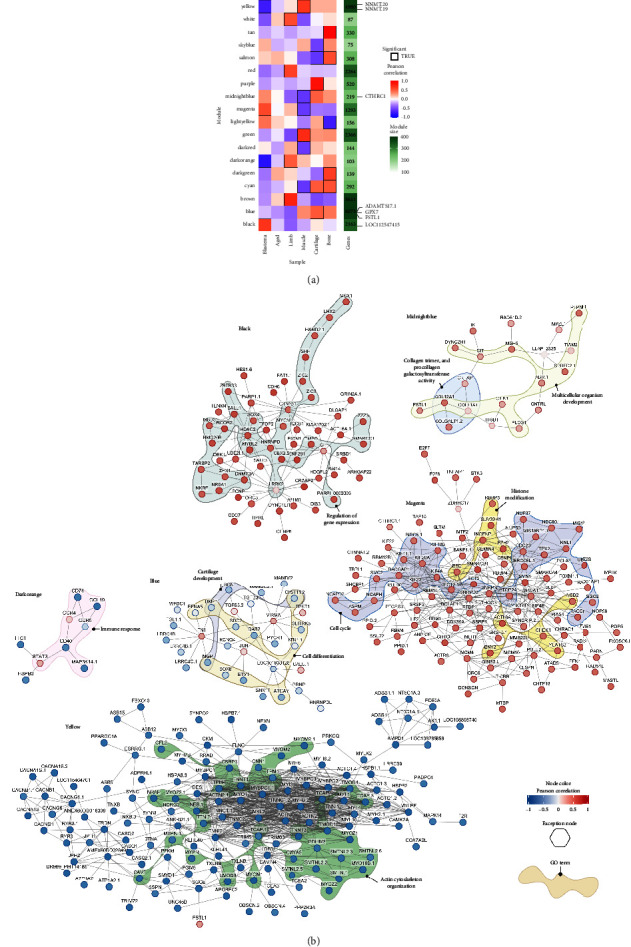
Gene coexpression analysis of axolotl samples. (a) Module–trait correlation for the 18 significant coexpression modules identified. Tile color represents the module–trait Pearson correlation coefficient. Significant modules (*p* value < 0.05) are enclosed in a black box The green panel indicates the module size. (b) Overview of the coexpressed genes within the black, midnight blue, yellow, blue, dark orange, and magenta modules. Node color corresponds to the Pearson correlation coefficient for the blastema samples. Edges represent the putative protein–protein interactions predicted by STRING. Gene ontology terms associated with some nodes are highlighted with colored shapes. Exception nodes (genes not belonging to the module) are marked with a hexagon. Created with BioRender.

As an additional approach, we used coexpression network analysis to examine sets of genes that might be involved in regeneration and show similar transcriptional responses to our set of regeneration-related genes. This analysis incorporated muscle, cartilage, and bone samples from the study by Bryant [23], as well as two leg samples from Caballero-Pérez [24]. The inclusion of these datasets allowed for a more comprehensive and robust understanding of the genes that are coregulated and, thus, similarly responsive, in *A. mexicanum*. The resulting network comprised 35 gene modules, of which 18 modules showed significant associations (*p* value < 0.05 and absolute Pearson correlation coefficient > 0.5) with the evaluated conditions (Supporting Figure 1, Supporting Table 3). Four of the significant modules showed a strong correlation with the blastema condition (yellow, magenta, dark orange, and black). The yellow and magenta modules were also significantly associated with muscle samples. Genes involved in metabolic processes and muscle structure development are enriched in the yellow module, while the magenta module showed enrichment in genes associated with the cell cycle, DNA replication, and RNA splicing. The black module is exclusively associated with blastema and was enriched in genes associated with developmental processes and multicellular organism development. Notably, the dark orange module did not show any significant GO term association but is also associated with limb samples (Figure 4(a), Supporting Figure 2).

Although none of the modules showed a strong association with the aged samples, we identified a set of 19 genes that had a significant association (absolute module–trait correlation > 0.7 and *p* value < 0.05) with the trait (Supporting Table 4). Among these genes, the two with the strongest correlation were *AMEX60DD027179*, a nonannotated gene encoding a 127 amino acid protein of unknown function, and *PAFAH2*, a gene encoding platelet-activating factor acetylhydrolase isoform 2. Both genes were negatively associated with aged axolotls and belonged to the turquoise module, which exhibited enrichment in genes associated with the regulation of metabolic processes, organelle organization, and chromatin organization.

To gain further insight into the biological processes associated with each module, we used KeyPathwayMiner to construct PPI networks.  Figure 4(b) displays the modules that were significant for blastema and those that contain one or more of the previously identified regeneration-related genes. Network nodes are color-coded based on their gene–trait correlation for blastema, while the edges represent the predicted interactions between the proteins encoded by the genes. The colored shapes indicate the GO terms associated with the module nodes. *CTHRC1,* one of the regeneration-related genes, was found in the midnight blue module, which exhibited significance in muscle samples but had no enriched GO terms. The network revealed proteins associated with multicellular development and collagen trimer. The blue module contained three of the seven regeneration-related genes identified through differential expression analysis (*ADAMTS17*, *GPX7*, and *FSTL1*). This module displayed a strong correlation between cartilage samples and enrichment in genes associated with the regulation of developmental processes. The PPI network for this module showed enrichment of genes related to cartilage development (e.g., *BMP1* and *CHST11*) and cell differentiation (e.g., *SOX8*, *SDC2*, and *DAB2*). Furthermore, the dark orange module, which negatively correlated with blastema, showed enrichment in immune response-related proteins, with some members associated with *STAT3*. The magenta module included genes related to the cell cycle, particularly belonging to the kinesins family (*KIF*). Notably, genes related to histone modification were also observed, including the polycomb group members *EED* and *EZH2*, as well as *SUV39H1*, a histone lysine methyltransferase. Genes involved in the regulation of gene expression, including *HDAC2* (histone deacetylase), *DNMT3A* (DNA methyltransferase), and transcription factors such as *SOX4*, *MYCN*, and *SALL1*, were found in the PPI network for the black module, one of the modules closely associated with the blastema and containing the regeneration-related gene *LOC112547415.* Finally, the two *nicotinamide N-methyltransferase (NNMT)* genes were found in the yellow module, which was enriched with several proteins associated with the organization of the actin cytoskeleton, as well as various components of the cytoskeleton, such as troponin genes (*TNT*), actin (*ACTN*), myotilin (*MYOT*), and myosin (*MYL*). Interaction between *SGCE*, another protein from the cytoskeleton, and *FSTL1* was also observed in this module.

In summary, we identified gene modules associated with regeneration and genes with similar transcriptional responses to our set of regeneration-related genes. Notably, these identified modules included our proposed regeneration-related genes, as well as other genes potentially involved in regeneration. These results highlight the potential interplay between the regeneration-related genes we identified and other factors involved in axolotl limb regeneration, offering insights into the potential biological significance of the identified genes.

#### 3.1.3. The Regeneration-Related Genes Are Conserved in Vertebrates

**Figure 5:**
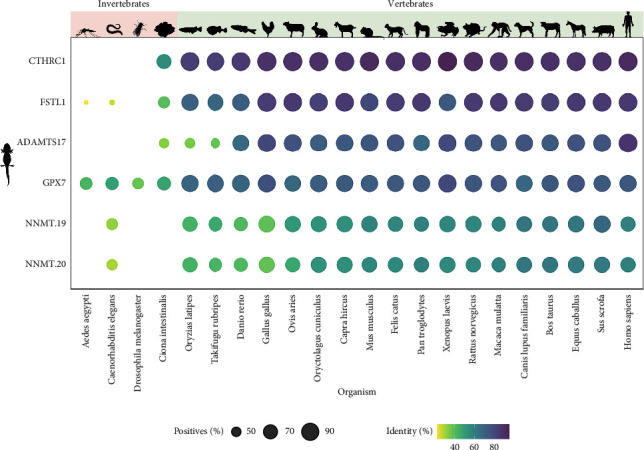
Conservation of regeneration-related proteins in *A. mexicanum*. The plot shows the percentage identity of each regeneration-related *A. mexicanum* protein (query) vs. its closest homolog in other selected animals (target). Bubble color indicates the percentage identity between the query and target protein, while the bubble size is proportional to the percentage of positives. Positives refer to amino acids in the subject sequence that are either identical to or have similar chemical properties as those in the query sequence. Missing values indicate that no similar proteins were found in the subject organism. Created with BioRender.

Through transcriptomic analysis, we identified a group of genes relevant to regeneration in *A. mexicanum*. To assess the functionality and presence of the proteins encoded by these genes in other organisms, we performed a homology search against the UniProtKB + Swiss-Prot database [44] using a selection of animals based on S. Hedges' comprehensive review of model organisms [45].  Figure 5 illustrates the identity between the proteins encoded by the regeneration-related genes and their closest homologs in each selected organism. The UniProt IDs for each sequence and the full set of results are given in Supporting Table 5.

It is noteworthy that all of the regeneration-related proteins in *A. mexicanum* show homology in vertebrates, although their presence in invertebrates is variable. Among the regeneration-related proteins, CTHRC1 has the highest percentage of identity in vertebrates, while the NNMT proteins have lower conservation and a lower percentage of positives, indicating that only a fraction of the target protein matches the query sequences. However, FSTL1 and ADAMTS17 also demonstrate conservation in vertebrates but not in invertebrates. GPX7 is the only protein from the regeneration-related genes that is found in all of the organisms evaluated, albeit with a noticeably lower percentage of identity in invertebrates compared to vertebrates. Notably, the gene *LOC112547415* does not appear in the graph as it lacks an associated open reading frame, leading us to hypothesize its potential as a noncoding RNA.

**Figure 6:**
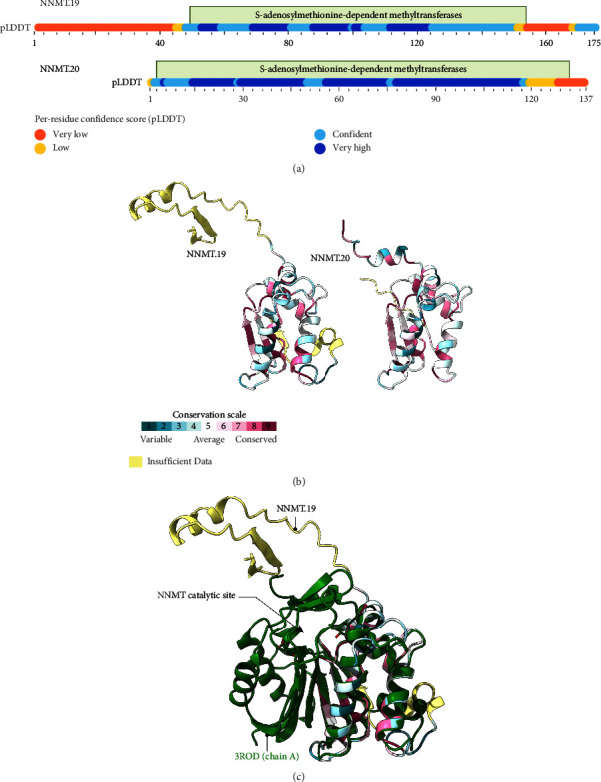
Sequence and conservation of *A. mexicanum* NNMT proteins. (a). Domain overview of *A. mexicanum* NNMT.19 and NNMT.20 proteins. The figures represent the sequence alignment of the proteins, with the scale corresponding to the number of amino acids in each protein. The predicted conserved domains are annotated with a green box. The per-residue confidence (pLDDT) for the AlphaFold models is represented by a bar. The complete structures can be found in Supporting Figure 3. (b) AlphaFold2 structure prediction for both NNMT proteins. The predictions were obtained using the monomer preset for AlphaFold, with templates. The structures are colored according to their sequence conservation as calculated by the ConSurf server. (c) Structural alignment between *A. mexicanum* NNMT.19 and an x-ray crystallography structure for human nicotinamide *N*-methyltransferase (NNMT) deposited in PDB (ID: 3ROD). Created with BioRender.

Subsequently, the 3D structure of the proteins encoded by each regeneration-related gene in *A. mexicanum* was modeled using AlphaFold2 [46], while sequence conservation was assessed using the Consurf [47] server and the UniRef90 database. The complete predictions and confidence scores can be found in Supporting Figure 3. In addition, the CDD [48] was used to identify conserved domains within the proteins, with the details summarized in Table 3. Regarding the NNMT proteins, they share 97% identity with each other (Supporting Figure 4), and both have an S-adenosylmethionine-dependent methyltransferases domain (Figure 6(a)). NNMT.19 also possesses an extra N-terminal domain that does not appear to be conserved, as indicated by the “insufficient data” label from ConSurf (Figure 6(b)). However, upon comparing NNMT.19 with a previously published crystallographic structure of human NNMT [50] it became evident that both axolotl proteins possess an incomplete catalytic domain, suggesting that only half of the protein is present in axolotls (Figure 6(c)), consistent with the low percentage of positive amino acids found previously.

**Figure 7:**
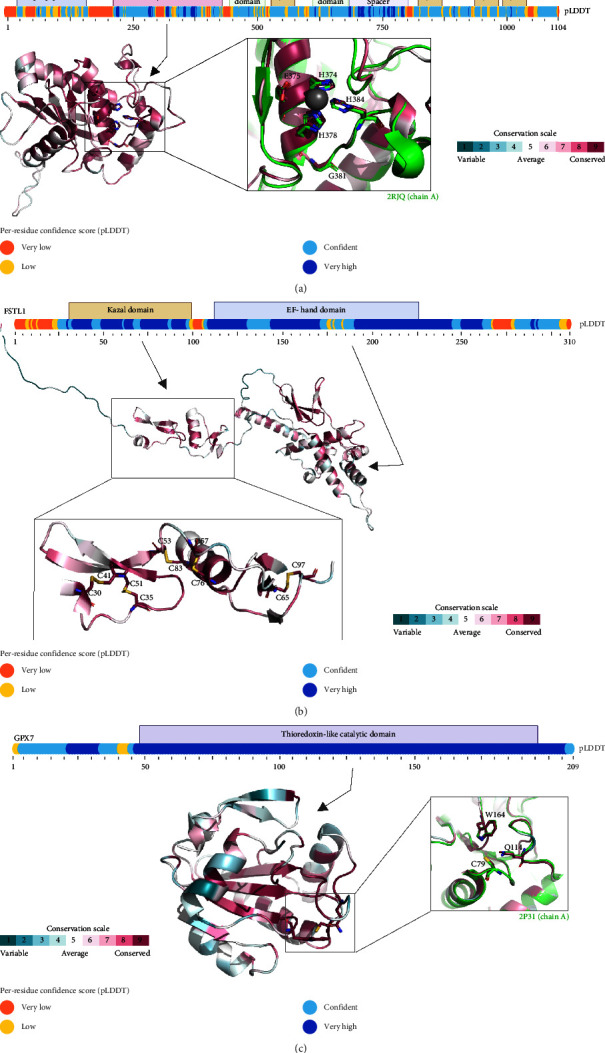
Conservation of ADAMTS17, FSTL1, and GPX7 proteins in *A. mexicanum*. Structures were predicted with AlphaFold2, and the conservation analysis was performed using the ConSurf server. (a) Domain overview and predicted structure of the catalytic domain of *A. mexicanum* ADAMTS17. Residues are colored according to their degree of conservation. The inset shows the structural alignment of the catalytic sites of *A.mexicanum* ADAMTS17 and *H. sapiens* ADAMTS5 (PDB: 2RJQ, green). The per-residue confidence (pLDDT) for the AlphaFold models is represented with a bar. The complete structures can be found in Supporting Figure 3. (b) Predicted domains and structure of *A. mexicanum* FSTL1. Residues are colored according to their degree of conservation. The inset shows the conservation of the cysteine bonds required for FSTL1 folding. The colored bar represents the per-residue confidence (pLDDT) for the AlphaFold model. (c) Domain overview and predicted structure of the catalytic domain of *A. mexicanum* GPX7. Residues are colored according to their degree of conservation. The inset shows the structural alignment of the catalytic sites of *A. mexicanum* GPX7 and *H. sapiens* GPX7 (PDB: 2P31, green). The colored bar represents the per-residue confidence (pLDDT) for the AlphaFold model. Created with BioRender.

ADAMTS17 from *A. mexicanum* has several conserved domains, including a catalytic domain [51] with the zinc-binding HExxHxxGxxH consensus motif, characteristic of the catalytic site of the ADAMTS family of metalloproteases [52–54]. It also contains a propeptide from the reprolysin family, two cysteine-rich regions, and several TSP-1 motifs. A comparison with the crystallographic structure of human ADAMTS5 [55], an enzyme of the same family, shows that the catalytic residues of the axolotl's ADAMTS17 are present and that the sequence around them is conserved, suggesting that this protein could be catalytically active (Figure 7(a)).

On the other hand, two domains were identified for FSTL1: a Kazal-type serine protease inhibitor and an EF-hand domain. The ConSurf analysis shows that FSTL1 from *A. mexicanum* is highly conserved. Notably, a set of disulfide bonds with high conservation, together with the Kasal domain, corresponds to the follistatin-like 1 (Fstl1-FK) domain described by Li et al. [56] (Figure 7(b)).

GPX7 has only one thioredoxin-like domain; however, structural analyses revealed that the catalytic site and residues of the protein are conserved in *A. mexicanum*. A comparison between the catalytic site of *H. sapiens* GPX7 [51] and the predicted structure for *A. mexicanum* GPX7 shows that the residues W164, C79, and Q114, which are required for GPX7 activity, are also present in our prediction (Figure 7(c)).

**Figure 8:**
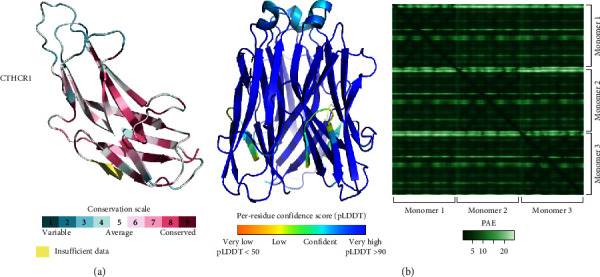
CTHRC1 conservation in *A. mexicanum*. (a). Complete predicted structure for *A. mexicanum* CTHRC1. The 3D structure was predicted using AlphaFold2, with residues colored according to their degree of conservation as calculated by the ConSurf server. (b) Heterotrimer 3D model prediction for CTHRC1. The prediction was obtained with AlphaFold2 multimer, with the residues colored according to the per-residue confidence score. The right panel shows the predicted aligned error for the trimer. Low values indicate the higher confidence in the residue–residue interaction. Created with BioRender.

Finally, no conserved domain was identified for the CTHRC1 protein; however, structural prediction and conservation analysis suggest its preservation (Figure 8). CTHRC1, also known as collagen triple helix repeat containing-1, is a secreted protein involved in osteogenesis and bone remodeling processes and typically exists as a homotrimer [57]. Using the AlphaFold multimer model, we obtained a high confidence structure for the axolotl CTHRC1 trimer. Taken together, our results suggest that the regeneration-related genes highlighted in this study encode proteins that are conserved in other vertebrates, allowing us to infer their function based on their homologs.

## 4. Discussion

*A. mexicanum*, commonly known as axolotl, is a salamander that captivates scientists due to its exceptional characteristics [58]. Unlike other amphibians, axolotls exhibit neoteny, retaining larval features while achieving sexual maturity [1]. However, their most remarkable attribute is their ability to regenerate nearly all tissues and organs, setting them apart from other vertebrates. Despite descriptive and histological studies shedding light on tissue regeneration in *A. mexicanum*, the underlying molecular mechanisms remain enigmatic [12, 14, 20]. Comprehensive studies of the molecular mechanisms involved in axolotl regeneration are of particular interest to the field of regenerative medicine, with potential implications for human therapies [30, 59]. To gain a broader understanding of the genetic basis of axolotl tissue regeneration, we conducted a transcriptomic study on a group of *A. mexicanum* from the CIBAC, a center dedicated to preserving the native axolotl population in Lake Xochimilco, Mexico City. Notably, these specimens were bred under conditions closely resembling their natural environment, reducing confounding factors that may be encountered in laboratory-raised counterparts [60, 61].

We began by examining samples from amputated limbs and the blastemas that form in response to injury. Blastema formation is triggered by signals from surrounding tissues that induce the dedifferentiation of nearby cells into a proliferative and multipotent state. This organ is crucial for regeneration in axolotls, and, because of that, is subject to complex transcriptomic regulation [19, 62]. Employing next-generation RNA sequencing, we conducted differential expression analysis between blastemas and juvenile limbs, comparing our results to a previously published dataset to identify shared expression patterns. Additionally, we generated a predicted interactome and performed GO annotation for the axolotl proteome to further describe the putative functions of the genes identified.

Taken together, our findings suggest a global downregulation of genes associated with muscular tissue and anatomical development during limb regeneration, consistent with the cellular dedifferentiation that takes place in the blastema [63]. However, a few genes related to development, such as *TBX4* [64], *BMP2* [65], and *KAZALD1* [23], as well as wound healing, such as *SALL4* [66], were upregulated in blastemas. Furthermore, genes associated with the regulation of cell differentiation, such as *WNT5A* and *WNT5B*, exhibited increased expression. *WNT5A* and *WNT5B* play established roles in bone morphogenesis, hematopoiesis, and cartilage homeostasis [67–69]. This suggests that, while tissue-specific genes are silenced during regeneration, a select few guide the development of the new limb.

In support of the aforementioned hypothesis, our gene coexpression network analysis revealed two modules positively associated with blastema samples. These modules contained several proteins involved in histone modification and DNA methylation, including *DNMT3A*, *HDAC2*, *SUV39H1*, *KDM3A*, and the Polycomb group members *EED* and *EZH2*. Epigenetic regulation has been shown to be crucial for tissue regeneration in axolotls; the pharmacological inhibition of DNA methyltransferases causes impairments in blastema formation [70], and histone deacetylase inhibitors inhibit regeneration [71]. The Polycomb group is also known for its role in embryonic development and tissue differentiation, suggesting its likely importance in axolotl tissue regeneration due to its evolutionary conservation [72].

Intriguingly, we also observed upregulation of genes associated with extracellular matrix organization in the blastema compared to control tissue, while cell adhesion factors were predominantly downregulated. This may be linked to the extracellular matrix degradation (histolysis) that occurs at the amputation site, facilitating cell migration, differentiation, and blastema formation [73]. Among the main players in this tissue remodeling process are metalloproteases, with several, such as *MMP13*, *MMP19*, *ADAM8*, and *ADAMTS17*, found to be upregulated in the blastema [74]. Furthermore, genes involved in angiogenesis and wound healing, including tenascin (*TNC*), fibronectin (*FN1*), and thrombospondin-2 (*THBS2*), are also upregulated. Alongside the overall downregulation of myosin and actin proteins, these findings indicate the extensive histolysis and angiogenesis process occurring in the tissue surrounding the blastema [75, 76].

The significance of the extracellular matrix becomes evident in the analysis of samples collected from aged axolotls that lacked regenerative capabilities. Notably, we detected the downregulation of several collagens and ribosomal proteins in aged limbs, indicative of impaired cell–cell interactions and extracellular matrix composition, commonly associated with aging [77, 78]. Processes such as osteogenesis, proliferation, and differentiation are influenced by extracellular matrix stiffness [79–81], making the reduced collagen expression in aged organisms particularly intriguing. This leads us to hypothesize that extracellular matrix organization plays a central role in axolotl regeneration. Further investigation into the changes occurring in the extracellular matrix during blastema formation and limb regeneration in axolotls could provide valuable insights into the regulatory networks involved in this process.

A comparative analysis between aged limbs and blastemas in juvenile specimens postamputation led us to the identification of a set of genes that may be required for initiating the regeneration process. Among these, seven genes were pinpointed, including one putative lncRNA (*LOC112547415.222*) and six coding genes. A blast search against model animals demonstrated homologous counterparts of the six coding proteins in vertebrates, indicating their potential importance. In particular, four regeneration-associated genes (*FSTL1*, *ADAMTS17*, *GPX7*, and *CTHRC1*) exhibited high expression levels in regenerating tissue but were underexpressed in aged axolotls. Interestingly, *ADAMTS17* is underexpressed in 8-year-old axolotls compared to juvenile axolotl limbs. This could suggest that the molecular mechanisms required for its transcriptional activity are impaired or that an epigenetic mechanism may be involved in this process [82]. This might partially explain why these organisms were unable to regenerate their limbs. Structural and conservation analyses further highlighted that these genes encode conserved proteins in vertebrates, which, together with structural predictions, allows us to infer their potential functions.

In this regard, ADAMTS17 is an extracellular metalloprotease involved in collagen processing, extracellular matrix degradation, cartilage cleavage, development, and angiogenesis. This protein has a conserved catalytic domain [52–54], suggesting it is catalytically active. Studies in *Adamts17* knockout mice showed that it is required for proper skeletogenesis and skeletal muscle development [83]. Mutations in ADAMTS17 are also linked to the Weill-Marchesani syndrome in humans, affecting connective tissue and leading to impaired vision, short stature, and musculoskeletal anomalies [84, 85]. Additionally, ADAMTS17 mutations have been implicated in short height and glaucoma in dogs [86] and humans [87]. Nevertheless, the precise function of this protein remains enigmatic. Studies have observed that a catalytically inactive ADAMTS17 interferes with fibrillin-1 secretion, resulting in elastic fiber abnormalities and intracellular collagen accumulation in fibroblasts from patients with Weill-Marchesani syndrome [88]. Another noteworthy protein involved in cell–matrix interactions is FSTL1, a secreted glycoprotein that participates in the regulation of the TGF-*β*, BMP, and Wnt pathways [89, 90]. In axolotls, the identification of two domains, a Kasal and an EF-hand domain, aligns with the architecture described in other organisms [56, 91]. FSTL1 has been found to be critical for tracheal and central nervous system development in mice, with *Fstl*1^−/−^ mice exhibiting cyanotic traits due to tracheal malformations [92, 93]. It is also involved in vascularization and vascular epithelial homeostasis maintenance [94, 95]. Overall, the observed downregulation of ADAMTS17 and FSTL1 in aged axolotls compared to juvenile limbs further highlights the importance of the extracellular environment during limb regeneration.

Furthermore, CTHRC1, involved in the TGF-*β* pathway, functions as a mediator of osteoblast–osteoclast communication and plays a role in osteogenesis and bone remodeling [96]. In axolotls, bone resorption mediated by osteoclast is important for the adequate integration of regenerated bone [97]. CTHRC1 also promotes angiogenesis by inhibiting collagen deposition and promoting cell migration [98]. Knockout mice for this gene showed reduced bone density and arthritis [99, 100], further suggesting the importance of this protein in bone and cartilage development.

GPX7, another protein with high expression in blastema but reduced levels in aged limbs, is a peroxidase, and its catalytic domain has been found to be conserved in axolotls. GPX7 is vital for oxidative stress resistance [101, 102]. Studies have linked low GPX7 levels to increased adipogenesis and fat accumulation in mice [103]. Interestingly, a recent study has observed that the bone marrow of aged axolotls has a higher fat content than that of its younger counterpart [30], which could be associated with the reduced GPX7 expression in aged axolotl limbs. Moreover, tissue regeneration in axolotls requires the production of reactive oxygen species [104], suggesting that enzymes involved in the regulation of oxidative stress are important for this process.

**Figure 9:**
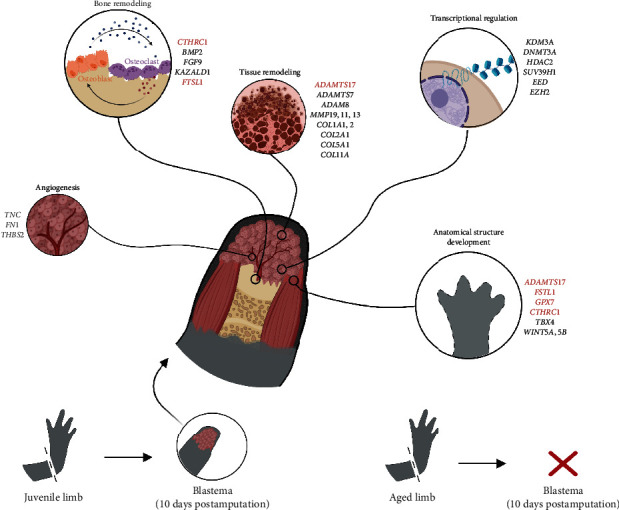
Graphical summary. The diagram displays key genes associated with blastema development during axolotl limb regeneration, along with the biological processes they are linked to. Genes considered vital for regeneration, highlighted in red, were identified by their absence in aged limbs that are incapable of regeneration. Created with BioRender.

Lastly, we also identified two NNMT proteins with high expression in aged axolotls but with decreased expression in the blastema. While the sequences of both proteins showed homology to other vertebrate NNMTs, we noticed that only half of the catalytic domain of the protein is present in our axolotl proteins. NNMT enzymes belong to the group of SAM-dependent methyltransferases [105], and they have been implicated in several epigenetic processes since SAM serves as the methyl donor used by DNA and histone methyltransferases. Consequently, high levels of NNMT cause a decrease in SAM availability, which leads to the inhibition of other methyltransferases [106, 107]. Although the functional status of the axolotl NNMT proteins we identified remains unclear, it is evident that these transcripts play a central role in aged axolotls, and further investigation is needed to unravel their mechanisms of action. These two transcripts were identified in the yellow coexpression module, displaying a strong negative correlation with blastema and enrichment in genes associated with metabolic processes, thereby accentuating their central role in regeneration and aged limbs. A graphical summary of our main findings is provided in Figure 9.

## 5. Conclusions

Our research provides a comprehensive overview of the changes in gene activity that occur during tissue regeneration in axolotls. By studying older axolotls, we discovered a group of genes that may be crucial for the regenerative abilities of *A. mexicanum*. Through an analysis of how these genes are preserved across different species, we suggest that they play critical roles in development, bone formation, and the organization of the extracellular matrix. Our results also indicate that the proteins produced by these genes exist in other vertebrates, suggesting that these genes could serve as a starting point for studying regeneration in other animals. Although our findings may not lead directly to therapeutic applications in human regenerative medicine, the insights gained from studying axolotl regeneration are invaluable to the field. Research has shown that mammalian transcription factors can trigger a regenerative response in axolotls, implying that similar regenerative mechanisms may exist in other organisms. Therefore, it is essential to identify the genes and factors responsible for initiating this process. Such knowledge has the potential to inspire innovative therapeutic strategies in human regenerative medicine in the future. In conclusion, our findings emphasize the importance of understanding the genes at work in *A. mexicanum*, providing valuable insights into the molecular processes behind tissue regeneration and the impact of aging on regenerative abilities.

## Figures and Tables

**Table 1:** Analysis of previously published samples.

SRA accession	Sample type	Citation
SRR2885267	Bone	[23]
SRR2885268	Bone	
SRR2885269	Bone	
SRR2885270	Forearm cartilage	
SRR2885271	Forearm cartilage	
SRR2885273	Forearm cartilage	
SRR2885553	Distal blastema	
SRR2885591	Distal blastema	
SRR2885592	Elbow	
SRR2885593	Elbow	
SRR2885594	Forearm	
SRR2885595	Forearm	
SRR2885597	Hand	
SRR2885598	Hand	
SRR2885599	Hand	
SRR2885865	Proximal blastema	
SRR2885866	Proximal blastema	
SRR2885867	Skeletal muscle	
SRR2885868	Skeletal muscle	
SRR2885869	Skeletal muscle	
SRR2885870	Skeletal muscle	
SRR2885871	Upper arm	
SRR2885873	Upper arm	
SRR2885875	Upper arm	

SRR5042766	Front leg	[24]
SRR5042769	Rear leg	

**Table 2:** Annotation and putative function of the regeneration-related genes identified.

Gene ID	Gene Name	Homolog	Location		
			Chromosome	Start	End
AMEX60DD003517	*ADAMTS17*	*ADAMTS17* A disintegrin and metalloproteinase with thrombospondin motifs 17 *Terrapene carolina triunguis* NCBI Reference Sequence: XP_026504145.1	chr11p	81488489	82602596

AMEX60DD019607	*GPX7*	*GPX7* PREDICTED: glutathione peroxidase 7 *Latimeria chalumnae* NCBI Reference Sequence: XP_006006032.1	chr1q	1276741180	1277033638

AMEX60DD040253	*CTHRC1.1*	*CTHRC1* collagen triple helix repeat-containing protein 1 *Xenopus laevis* NCBI Reference Sequence: XP_018123683.1	chr5q	899669621	899687475

AMEX60DD046943	*FSTL1*	FSTL1 follistatin-like 1 *Rhinatrema bivittatum* NCBI Reference Sequence: XP_026504145.1	chr7p	70865121	71016956

AMEX60DDU001030024	*LOC112547415.222*	uncharacterized protein LOC112547415 *Pelodiscus sinensis* NCBI reference sequence: XP_025045344.1	C0173161	1	8438

AMEX60DD053665	*NNMT.19*	NNMT Indolethylamine *N*-methyltransferase-like *Microcaecilia unicolor* NCBI Reference Sequence: XP_030077372.1	chr9p	79243911	79434013

AMEX60DD053666	*NNMT*.20	NNMT Nicotinamide *N*-methyltransferase-like *Rhinatrema bivittatum* NCBI Reference Sequence: XP_029428629.1	chr9p	79380383	79403217

*Note:* The gene IDs correspond to the AmexT_v47 transcriptome. The homolog sequence listed is based on the closest match found in the NCBI reference database.

**Table 3:** Domain annotation for the regeneration-related *A. mexicanum* proteins.

Name	Gene ID	Transcript ID	*E*-value	Residue range	Domain
NNMT.20	AMEX60DD053666	AMEX60DD201053666.1	1.80e- − 48	5–133	ID: cl17173 S-Adenosylmethionine-dependent methyltransferases

NNMT.19	AMEX60DD053665	AMEX60DD301053665.1	8.28e − 41	52–155	ID: cl17173 S-Adenosylmethionine-dependent methyltransferases

ADAMTS17	AMEX60DD003517	AMEX60DD201003517.1	1.18e − 24	26–163	ID: pfam01562 Reprolysin family propeptide
			3.56e − 84	217–434	ID: cd04273 Zinc-dependent metalloprotease, ADAMTS_like subgroup
			7.95e − 23	451–518	ID: cl20316 ADAM cysteine-rich domain
			7.31e − 16	531–583	ID: smart00209 TSP-1 type 1 repeat
			1.26e − 08	615–684	ID: cl41950 ADAM cysteine-rich domain
			3.06e − 07	821–873	ID: pfam19030 TSP-1 type 1 domain
			3.10e − 10	937–982	ID: pfam19030 TSP-1 type 1 domain
			2.14e − 12	991–1040	ID: pfam19030 TSP-1 type 1 domain

FSTL1	AMEX60DD046943	AMEX60DD201046943.1	3.46e − 12	30–9	ID: cd01328 Kazal-type serine protease domain
			4.27e − 73	112–225	ID: cd16233 EF-hand

GPX7	AMEX60DD019607	AMEX60DD301019607.1	5.40e − 105	46–198	ID: cl00388 Thioredoxin-like catalytic residues

*Note:* The domain ID is from the Conserved Domain Database (CDD).

## Data Availability

The GO term information and PPI network built and used in this work can be explored in STRING using the organism identifier STRG0034MNQ or the link https://version-11-5.string-db.org/organism/STRG0034MNQ. Additionally, the gene annotation file, ppi predicted network, an AnnotationDbi OrgDb package, and a Txdb object with the gene names and associated GO terms can be accessed at the https://github.com/aylindmm/A.mexicanum GitHub repository. The raw sequencing data were deposited in NCBI's Gene Expression Omnibus under Accession ID GSE237864.

## Supporting Information

Supporting Information Supporting_Figures.pdf—Supporting figures 1 to 4.

Supporting Information Supporting_Table 1.xlsx—The log2FC and other statistics associated with the DEGs in the conditions evaluated.

Supporting Information Supporting_Table 2.xlsx—Results obtained for the enrichment analysis of the consensus DEGs in the blastema.

Supporting Information Supporting_Table 3.xlsx—This table contains the module membership and gene–trait correlation obtained with WGCNA for the set of *A. mexicanum* samples evaluated.

Supporting Information Supporting_Table 4.xlsx—This table contains the module membership and gene–trait correlation obtained with WGCNA for genes significantly correlated with the “aged” trait. An absolute Pearson correlation.

Supporting Information Supporting_Table 5.xlsx—Subject sequences retrieved by Blastp for each of the “Regeneration-associated genes.”
