# Supplementary material for: Key Proteins for Regeneration in A. mexicanum: Transcriptomic Insights From Aged and Juvenile Limbs
Source: Scientifica (Cairo). 2024 Nov 14;2024:5460694. doi: 10.1155/2024/5460694 (PMC11581807; doi:10.1155/2024/5460694)
Supplement: Supporting Information — Supporting_Figures.pdf—Supporting figures 1 to 4. [file 5460694.f1.pdf]

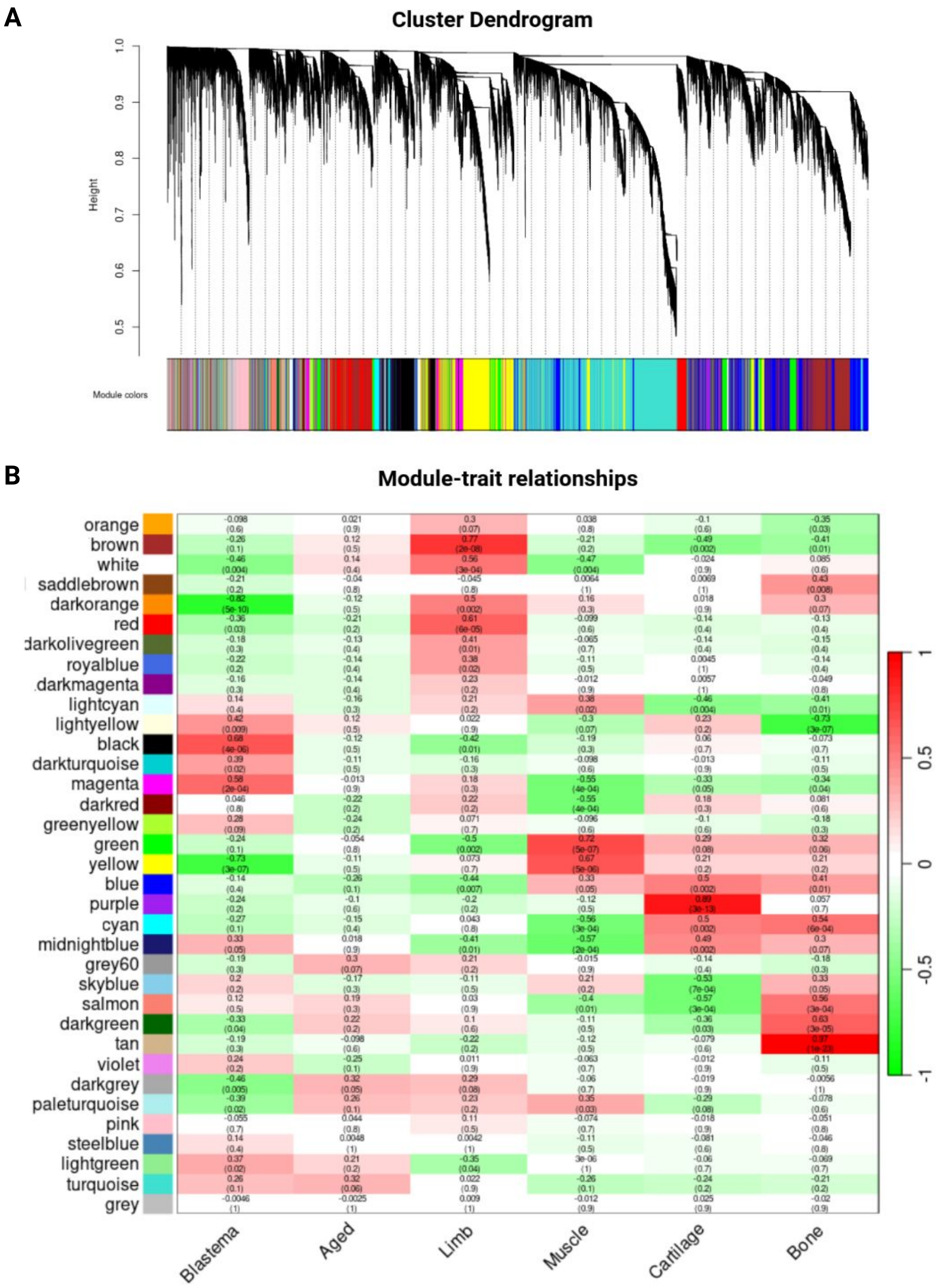


**Appendix Figure S1.** Gene Co-expression Network.

A Cluster Dendogram of genes and corresponding module color.

B Module-trait relationships for all the gene modules identified with the coexpression analysis. Tile color represents the Pearson Correlation coefficient.

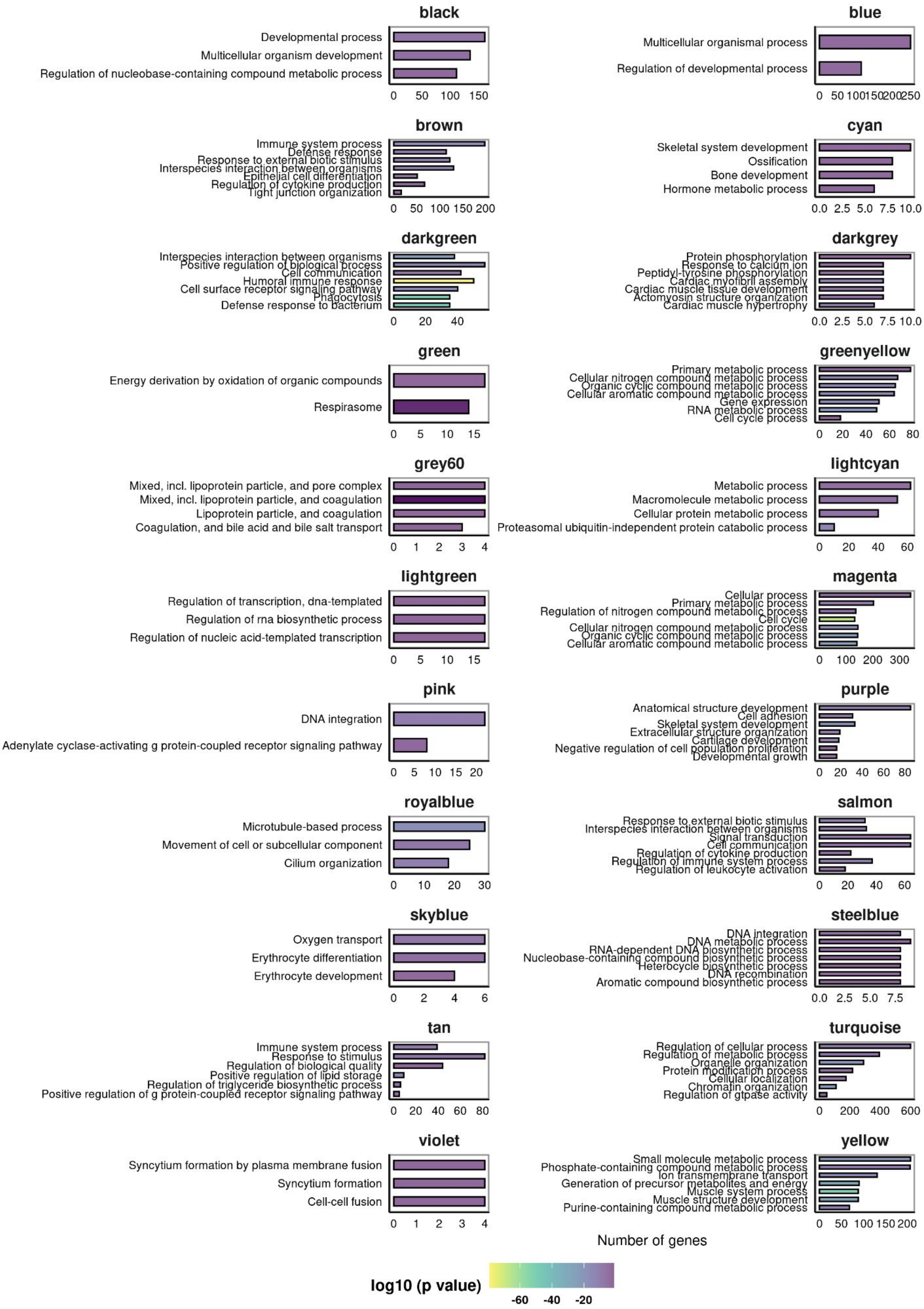


**Appendix Figure S2.** Gene Ontology terms associated to the gene modules from the co-expression network. The x axis represents the number of genes in each term found in the module. The bars are colored according to the p value of the enrichment analysis. Some of the modules from **Appendix Figure S1** are missing because they had no significant term associated.

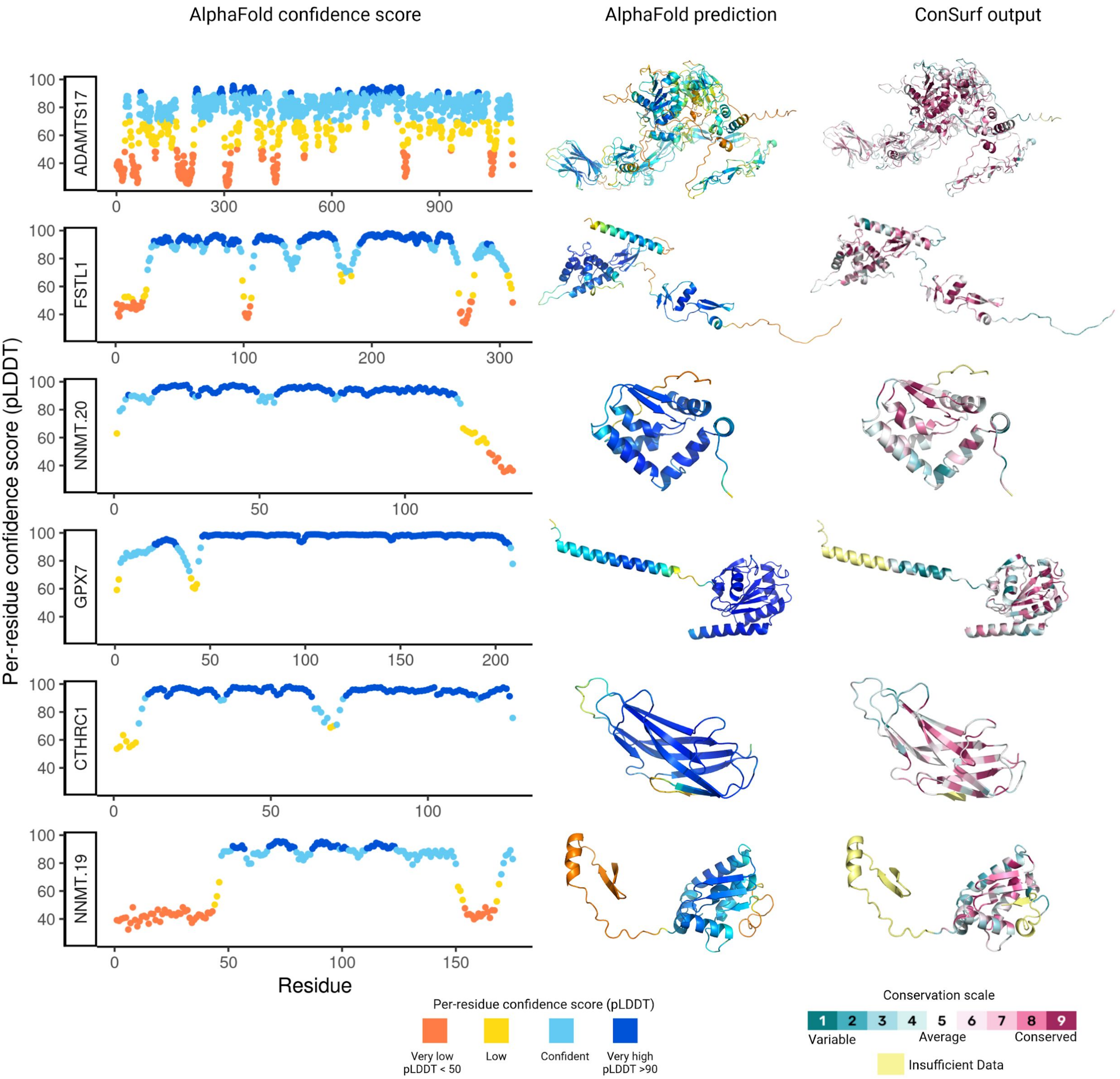


**Appendix Figure S3.** AlphaFold2 predictions for the regeneration-associated *A. mexicanum* proteins. The left panel shows the per residue confidence score (pLDDT) for the AlphaFold predictions, dots are colored according to their confidence level. The AlphaFold 3D structures in the middle panel are colored according to their per-residue confidence score. The same structures are presented in the left panel but colored according to the ConSurf conservation scale.

**A**

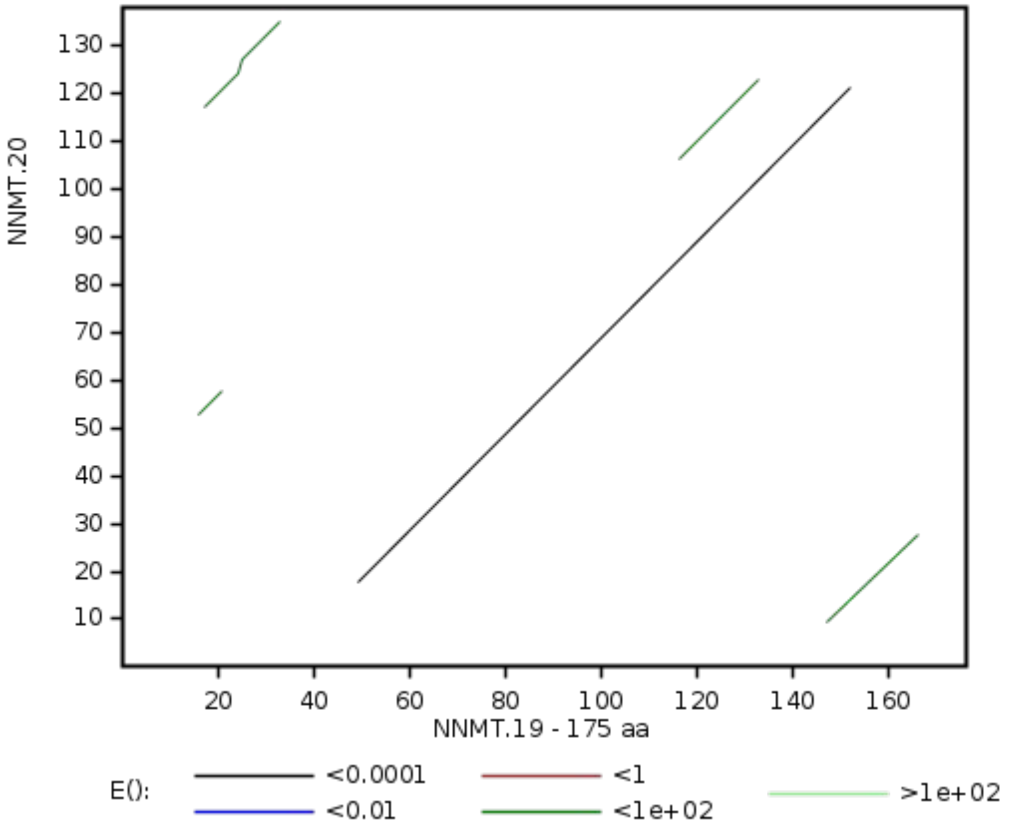


**B**

```
Waterman-Eggert score: 676;  215.1 bits; E(1) <  4.4e-61
97.1% identity (99.0% similar) in 104 aa overlap (49-152:18-121)

        50        60        70        80        90       100       110       120       130       140       150
NNMT.19 QSVLDTYYSESAGALVNDKYLEFVLKQLAKTFTAGVVEGDTLMDIGTGPTIYQLLSACEVFKEIIVTDYSAISRQEFEKWLKKDPGCFDWSPIVRYVCDLEGQR
        .. :::::::::::::::::::::::::::::::::::::::::::::::::::::::::::::::::::::::::::::::::::::::::::::::::::::
NNMT.20 RAYLDTYYSESAGALVNDKYLEFVLKQLAKTFTAGVVEGDTLMDIGTGPTIYQLLSACEVFKEIIVTDYSAISRQEFEKWLKKDPGCFDWSPIVRYVCDLEGQR
         20        30        40        50        60        70        80        90       100       110       120
```

**Appendix Figure S4.** Sequence alignment for A. mexicanum NNMT.19 and NNMT.20.
A Graphical depiction of the sequence identity between the two proteins. Line color indicates the E value for the suggested alignment. B Sequence of the region scored as the best non-identical alignment, this segment corresponds to the black line in panel a. The alignment was computed by LALIGN: [https://www.ebi.ac.uk/Tools/psa/lalign/](https://www.ebi.ac.uk/Tools/psa/lalign/)
